# Temporomandibular joint aging and potential therapies

**DOI:** 10.18632/aging.203332

**Published:** 2021-07-15

**Authors:** Yueying Zhou, Ming Xu, Sumit Yadav

**Affiliations:** 1 Hunan Key Laboratory of Oral Health Research and Hunan 3D Printing Engineering Research Center of Oral Care, Hunan Clinical Research Center of Oral Major Diseases and Oral Health, Xiangya Stomatological Hospital, and Xiangya School of Stomatology, Central South University, Changsha, Hunan, China; 2 UConn Center on Aging, UConn Health, Farmington, CT 06030, USA; 3 Department of Genetics and Genome Sciences, UConn Health, Farmington, CT 06030, USA; 4 Division of Orthodontics, UConn Health, Farmington, CT 06030, USA

**Keywords:** aging, TMJ, cellular senescence, senolytics

Degenerative disorders of the Temporomandibular Joint (TMJ) are the second most common chronic musculoskeletal disorder [1]. The degeneration of the TMJ in the affected individuals leads to severe pain, restricted joint mobility, and significant health cost (greater than 4 billion annually) to the society [1] Our previous animal and clinical study have demonstrated that aging is a significant risk factor for degenerative disorders of TMJ [2,3]. In our retrospective clinical study, we observed TMJ degeneration increases with every decade of life [3]. As an individual ages, there is a deterioration of the musculoskeletal health, including the osteochondral tissues of the TMJ [3]. The United States and the world population ages over the next several decades and the incidence of TMJ degenerative disorders are expected to rise substantially. Currently there are no effective clinical treatments for degenerative disorders of TMJ and total joint replacement becomes the only option. The treatment approaches being developed to treat joint cartilage and subchondral bone degeneration in aged individual is to replace a diseased or lost tissue with cartilage grafts or putative cartilage-forming cells, such as autologous chondrocytes or mesenchymal stem cells (MSCs), that infiltrate the joint during microfracture. However, clinical trials show that these approaches are not a long-term solution. Additionally, many stem cell therapies are still under investigation, and none of them are currently approved by FDA to alleviate the course of degenerative disorders. The barrier to success in these approaches is that they fail to produce replacement cartilage tissue that recapitulates the cellular composition, structure, and load-bearing capacity of normal joint cartilage.

The underlying mechanisms in age-related TMJ degeneration is largely unknown. Growing evidence suggests that retarding the fundamental aging process could simultaneously alleviate a range of age-related diseases and tissue dysfunction, which is now referred to as the Geroscience Hypothesis. Due to the high prevalence and uniquely complex nature of the TMJ in aging, the geroscience-guided approach, by targeting the whole aging process, might be beneficial for alleviating TMJ degeneration with aging. Among a number of basic aging mechanisms, cellular senescence has been indicated as a promising target [4]. Cellular senescence refers to the essentially stable growth arrest that occurs when cells experience various stresses. With aging or other pathological conditions, senescent cells accumulate in multiple tissues including joints [5], secreting a variety of pro-inflammatory cytokines, chemokines, and proteases, termed the senescence-associated secretory phenotype (SASP) [6]. It has been postulated that senescent cells accumulate in the musculoskeletal tissue with aging in a temporal and spatial pattern, which impair the function and exacerbate age related pathologies of the musculoskeletal tissue. The senescence of the chondrocytes could be an age dependent process due to both intrinsic and extrinsic factors. The release of pro-inflammatory cytokines by the senescent cells contributes to the catabolic degradation of the extracellular matrix in the osteochondral tissues of the TMJ. Thus, it might be feasible to alleviate musculoskeletal disorders of aging by targeting senescent cells.

Indeed, in our recently published research, we observed that intermittent oral administration of Dasatinib (D) + Quercetin (Q) reduced senescent cells, preserved mandibular condylar cartilage thickness, improved subchondral bone volume and turnover, and reduced histopathological score in TMJs from both male and female aged mice, whereas D+Q had little effect on the TMJ in young mice [7]. D+Q is the first senolytic cocktail shown to specifically kill senescent cells, and has been shown to mitigate a range of age-related tissue dysfunctions [4]. Our findings expanded the benefits of D+Q to the joint health in the older individuals.

In summary, senolytic drugs showed promising benefits on TMJ health in aging populations. The results of our studies might open avenues to develop pharmaceutical interventions targeting cellular senescence to improve the regeneration of the osteochondral tissues of the aged TMJs, and possibly other joints. It is necessary to further investigate the molecular and cellular mechanisms by which senescent cells lead to joint degeneration with aging. Notably, although D+Q has been shown to be relatively safe and well tolerated in older human subjects by a small clinical trial [8], and multiple clinical trials are ongoing for several human diseases, rigorous large-scale clinical trials are absolutely critical before these findings can be translated into human use.
